# References for Small Fluorescence Quantum Yields

**DOI:** 10.1007/s10895-024-03729-2

**Published:** 2024-05-15

**Authors:** Mahbobeh Morshedi, Simon L. Zimmermann, David Klaverkamp, Peter Gilch

**Affiliations:** Institut für Physikalische Chemie, Heinrich-Heine-Universität Düsseldorf, Universitätsstr. 1, 40225 Düsseldorf, Germany

**Keywords:** Fluorescence quantum yield, Time resolved spectroscopy, Strickler-Berg analysis, Thymidine, Dibenzoylmethane, Malachite green chloride

## Abstract

Three compounds with fluorescence quantum yields in the range of 10^− 5^ to 10^− 4^ and emission spectra covering the UV/Vis spectral range are suggested as new references for the determination of small fluorescence quantum yields. The compounds are thymidine (dT) in water, dibenzoylmethane (DBM) in ethanol, and malachite green chloride (MG) in water, representing the blue, green, and red regions of the spectrum, respectively. All compounds are easily handled, photostable, and commercially available. Furthermore, these compounds exhibit a mirror-image symmetry between their absorption and fluorescence spectra. This symmetry, along with closely aligned fluorescence excitation and absorption spectra, confirms that the observed emissions originate from the compounds themselves. The fluorescence quantum yields were determined via a relative approach as well as Strickler-Berg analysis in conjunction with time resolved fluorescence spectroscopy. Within the respective error margins, the two approaches yielded identical results.

**Supplementary Information:**

The online version contains supplementary material available at 10.1007/s10895-024-03729-2.

## Graphical Abstract

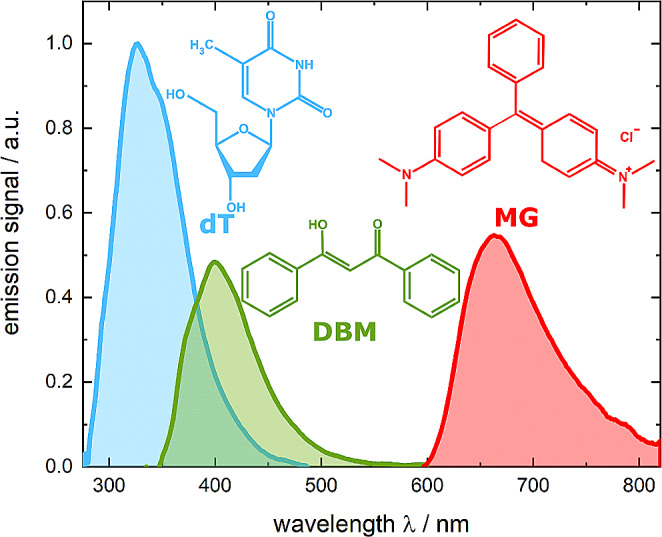

## Introduction

The fluorescence quantum yield *Ф*_fl._ is a key parameter of molecular chromophores. It is given by the ratio of the number of photons emitted *via* fluorescence to the number of photons absorbed by a chromophore. From this definition, it is immediately clear that the yield *Ф*_fl._ is a crucial figure of merit in fluorescence applications. For instance, optical brighteners [1, 2], ingredients of text markers [3, 4], emitters for organic light-emitting diodes (OLEDs) [5, 6], and fluorescence labels for modern microscopy [7] ought to feature yields *Ф*_fl._ close to one. Furthermore, based on these yields, reliable estimates of lifetimes of primary molecular excitations can be made [8]. This gives important first glimpses on the photophysics and photochemistry of a chromophore and facilitates the planning of time resolved spectroscopy [9]. Additionally, precise determinations of the yield *Ф*_fl._ are essential for the quantitative interpretation of Förster resonance energy transfer (FRET) experiments [10, 11].

It is, thus, no surprise that various techniques to determine these yields have been developed. They can be divided into absolute and relative ones. In absolute calorimetric approaches [12, 13], small temperature increases caused by the illumination of a sample are recorded. The temperature increase is approximately proportional to 1-*Ф*_fl._. Calorimetric determinations can mostly be found in the pre-1990s literature. Nowadays, absolute determinations often make use of integrating spheres [14, 15]. With these spheres, signals proportional to the emitted and absorbed light fluxes are recorded. The ratio of these quantities is approximately equal to the yield *Ф*_fl._. Contrary to the absolute methods, for which not so common set-ups are required, relative determinations rely on a widely-used instrument, namely a fluorescence spectrometer. In a relative determination, the spectrally integrated fluorescence signal of a sample is compared with the respective integral of a suitable reference (cf. Equation (4)) [16, 17]. Often solutions of dye molecules serve as a reference [18]. References based on the Raman scattering of neat solvents have also been reported [19].

Therefore, reference materials with approved and certified fluorescence quantum yields are particularly important for many users of fluorescence methods. According to Brouwer [20], the majority of these reference compounds have high fluorescence quantum yields *Ф*_fl._, commonly exceeding 0.5. Many chromophores exhibit yields *Ф*_fl._ many orders of magnitude smaller. Based on the relation (Eq. (1)) between the yield *Ф*_fl._ and rate constants for the radiative (*k*_*rad*_) as well as the non-radiative decay (*k*_*nr*_)

1 $${\varPhi }_{fl}=\frac{{k}_{rad}}{{k}_{rad}+{k}_{nr}}$$

a lower boundary for this yield can be estimated. We hereby restrict ourselves to organic chromophores with allowed lowest energy singlet transitions. For such chromophores, the radiative rate constant *k*_*rad*_ is of the order of 10^8^ s^− 1^ [9]. Internal conversion (IC) [21, 22], intersystem crossing (ISC) [21, 23–25], excitation energy (EET) [26] as well as electron transfer (ET) [27, 28], and photochemical transformations [28] can lead to non-radiative decays. The upper limit for rate constants of all of these processes results from nuclear motions [21, 29]. Characteristic frequencies of these motions are the ones of molecular vibrations with values of ∼ 10^13^-10^14^ s^− 1^ [21, 30]. Thus, molecules with allowed transitions and ultrafast non-radiative decays, i.e., *k*_*nr*_=10^13^-10^14^ s^− 1^, will exhibit fluorescence quantum yields *Ф*_fl._ of the order of 10^− 6^-10^− 5^. Obviously, for molecules with (partially) forbidden transitions, even smaller values may be found. Fluorescence quantum yields *Ф*_fl._ many orders of magnitude smaller than one were indeed often observed experimentally. DNA and RNA bases, for instance, feature yields of ∼ 10^− 4^ [31]. Photoreactive molecules like *trans*-azobenzene (∼ 10^− 7^ [32]), *cis*-stilbene (∼ 10^− 5^ in acetonitrile [33]), and rhodopsin (∼ 10^− 5^ [34]) exhibit even smaller yields. Triplet sensitizers like xanthone (∼ 10^− 4^ in ethanol [35]) and thioxanthone (∼ 10^− 5^ in cyclohexane [36]) are also examples for chromophores with very small yields. These examples emphasize the important role of compounds with small fluorescence quantum yields in diverse fields and underscore the need for establishing new reference materials to precisely quantify these yields. Relative determinations of such small fluorescence quantum yields are hampered by the predominance of references with yields of the order of one. As any quantitative comparison, the relative determination of these yields is facilitated if sample and reference exhibit similar signal strengths [37]. This similarity ensures that the measured fluorescence intensities are within the dynamic range of the instrument, thereby reducing errors associated with instrument sensitivity and signal detection limits [38, 39].

Here, we suggest and characterize three references with yields in the range of 10^− 5^-10^− 4^ and emission spectra covering the UV/Vis range. Fluorescence quantum yields *Ф*_fl._ of the compounds were determined with the relative approach as well as utilizing the relation between this yield, the radiative rate constant *k*_*rad*_, and the fluorescence lifetime ${\tau }_{fl.}$ (Eq. (2)) [8],

2 $${\varPhi }_{fl}={k}_{rad}{\tau }_{fl.}$$

The radiative rate constant *k*_*rad*_ was retrieved from absorption and fluorescence emission spectra *via* the Strickler-Berg relation (cf. Equations (5) and (6)) [40, 41]. This relation can be applied provided that the same pair of electronic states is involved in the absorption and the emission process. Furthermore, the Condon approximation must be valid [42]. A mirror-image relationship between absorption and emission spectra is an indicator that these conditions are fulfilled [21]. Thus, molecules not obeying this relation were discarded. The fluorescence lifetime ${\tau }_{fl.}$ was measured using fluorescence Kerr gating [36, 43]. For the weakly fluorescent samples considered here, signals of impurities might surmount the ones of the nominal sample [44]. Matching absorption and fluorescence excitation spectra indicate that the fluorescence indeed (predominately) stems from the sample and not an impurity. Thus, for all samples this was investigated. In addition to these fundamental criteria, also practical ones were considered. Chromophores and solvents commercially available in high purities were selected. The chemical and photochemical stability of chromophores in the given solvent were also a criterium.

**Fig. 1:**
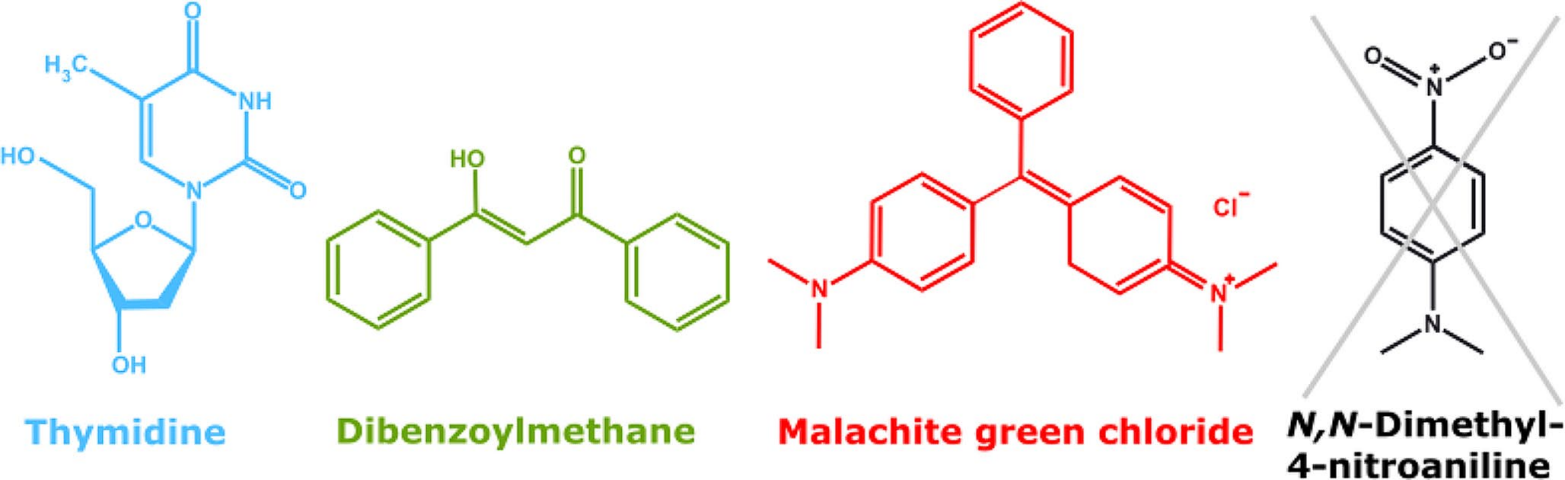
Chemical structures of thymidine, dibenzoylmethane, malachite green chloride, and *N, N*-dimethyl-4-nitroaniline. The first three molecules plotted in different colors are suggested as references, the last one was discarded

Based on these criteria, the following three chromophore/solvent combinations were identified and characterized. The combinations are thymidine (dT) in water, dibenzoylmethane (DBM) in ethanol, and malachite green chloride (MG) in water (see Fig. 1). In this order, they cover the blue, green, and red regions of the UV/Vis range.

For dT in water, previous determinations yielded a fluorescence quantum yield *Ф*_fl._ of the order of 10^− 4^ [45, 46]. For the other two chromophores, reported lifetimes in the range of 100 fs – 1 ps [47–51] suggested yields of the same magnitude. We will also report on a molecule, namely *N, N*-dimethyl-4-nitroaniline (DpNA), which was discarded as determinations based on the relative approach and the one based on the Strickler-Berg analysis in combination with a lifetime measurement did not match.

Following common practice in fluorescence spectroscopy and for ease of handling, solutions were *not* de-oxygenated. Oxygen quenching should essentially not affect yields *Ф*_fl._ of the compounds listed in Fig. 1. Assuming diffusion limited oxygen quenching (rate constant *k*_*q*_ ~10^10^ M^− 1^ s^− 1^ [9]) and inserting a typical concentration of dissolved oxygen ([O_2_] ∼ 10^− 3^ M [9]), one arrives at a time constant ${\tau }_{{O}_{2}}$ for oxygen quenching of the order of 100 ns. This is many orders of magnitude longer than the fluorescence lifetimes ${\tau }_{fl.}$ measured for the compounds in Fig. 1. Thus, oxygen quenching will not affect their fluorescence lifetimes, and according to Eq. (2), the yield *Ф*_fl._. However, oxygen quenching may affect the yields of the references employed in the relative determinations. The respective compounds feature fluorescence lifetimes ${\tau }_{fl.}$ in the 1–10 ns range which is closer to the lifetime ${\tau }_{{O}_{2}}$. To avoid systematic errors, references with reported fluorescence quantum yields for aerated solutions were employed.

## Experimental Section

### Samples

Thymidine (≥ 99.0%) (CAS ID: 50-89-5), L-tyrosine (≥ 99.8%), and malachite green chloride (≥ 90.0%) (CAS ID: 569-64-2) were purchased from Sigma-Aldrich, dibenzoylmethane (99.01%) (CAS ID: 120-46-7) from BLDpharm, coumarin-1 (99.9%) from Acros Organics, rhodamine 101 from Radiant Dyes Laser & Accessories GmbH, *N, N*-dimethyl-4-nitroaniline (≥ 98%) from Tokyo Chemical Industry, water (HPLC gradient grade) from Fisher Chemical, ethanol (≥ 99.8%) from Sigma-Aldrich, and acetonitrile (HPLC gradient grade) from Chem Solute. All measurements were performed at room temperature (∼ 20 °C).

### Steady State Measurements

Steady state absorption spectra were recorded using Lambda 19 and 1050 + spectrometers from Perkin Elmer. Fluorescence spectra were measured with a FluoroMax-4 (Horiba Scientific). Spectra were corrected for the spectral sensitivity of the instrument. For all steady state measurements, fused silica cells (from Hellma Analytics) with a path length of 1 cm were employed. In the steady state fluorescence experiments, the sample absorption at the excitation wavelength was kept at $\le$0.05 for a path length of 1 cm. The small absorption values avoid inner filter effects, i.e. re-absorption of emitted fluorescence, and more importantly ensures a linear scaling between the absorption values and the fluorescence signals. Equation (4) for the relative determination of the fluorescence quantum yields is based on such a scaling. The excitation and emission bandpasses were set to 5 nm for the steady state measurements of all compounds and their respective references. Also, the other settings of the fluorescence spectrometer were identical for samples and references. For the relative determination of the fluorescence quantum yields *Ф*_fl._ of DpNA in acetonitrile, dT in water, DBM in ethanol, and MG in water, the following references were used, respectively: coumarin 1 (C-1) in de-oxygenated water (${\varPhi }_{fl}^{r}$ = 0.055, note that the value is not significantly affected by oxygen [52]), tyrosine (Ty) in aerated water (${\varPhi }_{fl}^{r}$ = 0.21 ± 0.01 [53, 54]), C-1 in de-oxygenated water (${\varPhi }_{fl}^{r}$ = 0.055 [52]), and rhodamine 101 (Rh 101) in aerated ethanol (${\varPhi }_{fl}^{r}$ = 0.913±0.046 [55]). The samples and their respective references were excited at wavelengths close to their absorption maxima, while care was taken to ensure the coverage of their entire emission spectra. The region (~5 nm) around the excitation was avoided. The fluorescence spectra of all samples and their respective references were corrected for the Raman effect by subtracting a suitably scaled spectrum of the solvent, which was recorded under identical conditions.

### Time Resolved Fluorescence Measurements

The setup was described in detail elsewhere before [36, 43]. A 1 kHz Ti:Sa laser amplifier system (Coherent Libra) was employed as a pulse source. Its output has a wavelength of 800 nm and a pulse duration of ∼ 100 fs (full width half maximum, FWHM). For the experiment on DpNA in acetonitrile, the excitation wavelength was set to 400 nm. To this end, a portion of the output was converted (in a BBO crystal type I, 29°, 1 mm) to a wavelength of 400 nm by frequency doubling. The beam had an energy per pulse of 1 μJ at the sample location. For the experiments on dT in water and DBM in EtOH, the excitation wavelength was set to 266 nm. To this end, a portion of the output was initially converted (in a BBO crystal type I, 29°, 1 mm) to a wavelength of 400 nm by frequency doubling. Subsequently, the sum frequency was generated (in another BBO crystal, type II, 55.5°, 0.5 mm) to obtain a wavelength of 266 nm from the frequency doubled and the fundamental beam. At the sample location the beam had a diameter of 80 μm (FWHM) and a pulse energy of 1 μJ. For the measurement on MG in water, the excitation wavelength was tuned to 580 nm. To this end, a part of the output was directed to a TOPAS-White non-collinear optical parametric amplifier system. The TOPAS was set to generate pulses peaking at 580 nm with an energy of 0.9 μJ per pulse. The generation of the gate pulses and the operation of the Kerr gate followed the description in ref. [36]. The width of the instrumental response function (IRF), as obtained from Raman scattering of the solvent, was about 250, 270 and 220 fs (FWHM) for 400, 266 and 580 nm excitation light, respectively. For the experiment on DpNA in acetonitrile, the integration time was set to 1 s. Between − 5 and 3 ps, the delay time was varied linearly in 60 steps. A total of 13 scans were averaged. For the measurement on dT in water, the integration time for each spectrum was set to 1 s. One scan consisted of 30 equidistant steps between − 2 and 3 ps. A total of 14 scans were averaged. For the experiment on DBM in EtOH, the integration time of 2 s was set for each spectrum. In each scan, there were 60 equidistant steps on a linear time axis from − 2 to 3 ps. 22 scans were averaged. For the experiment on MG in water, the integration time was set to 1 s. Between − 2 and 3 ps, the delay time was varied linearly in 60 steps. A total of 110 scans were averaged. The solutions were circulated through a flow cell (custom made QX, Hellma Analytics) with a path length of 1 mm by a peristaltic pump (REGLO Analog MS-2/8 from ISMATEC®). Signals on solutions of DpNA, dT, DBM, and MG (concentrations of ∼ 0.5 mM, ∼ 1 mM, ∼ 1.3 mM, and ∼ 70 μM, respectively) as well as the neat solvent were recorded. The solvent contributions were subtracted after proper scaling. All time resolved spectra were corrected for the spectral sensitivity of the instrument.

### Data Analysis

Time resolved data sets *S(λ, t)* were analyzed globally with a multi-exponential fit function convoluted with the instrumental response function (IRF),

3 $$S\left( {\lambda ,t} \right) = IRF \otimes \sum\limits_{i = 1}^n {{S_i}} \left( \lambda \right) \cdot {e^{ - \frac{t}{{{\tau _i}}}}}.$$

The fit yields time constants ${\tau }_{i}$ and decay associated spectra *S*_*i*_ (λ) (DAS) [56, 57].

### Estimates of Error Margins

For the determination of the statistical error of the yield ${\varPhi }_{fl}^{rel}$ (measured by the relative method) and absorption coefficient (*ɛ*), multiple independent measurements were performed and the mean value was calculated [58]. The corresponding error margins denote the standard deviations from the mean [58]. The error margins of the reference yield ${\varPhi }_{fl}^{r}$, if available, were accounted for by error propagation. To determine the error of the radiative rate constant *k*_*rad*_, the error of the respective integrals (see Eq. (5)) entered an error propagation analysis [58]. The error margins in the time constants ${\tau }_{fl.}$ represent the deviations of the fit from the fluorescence decay data. These margins are determined through exhaustive search error analysis, utilizing the chi-squared (χ^2^) statistics to evaluate the quality of the fit by taking into account the correlation among all the fit parameters [59]. The quoted error in the fluorescence quantum yield ${\varPhi }_{fl}^{SB}$ (determined through the time resolved method) reflects the propagated errors associated with both the radiative rate constant *k*_*rad*_ and the fluorescence lifetime ${\tau }_{fl.}$.

## Results and Discussion

**Fig. 2:**
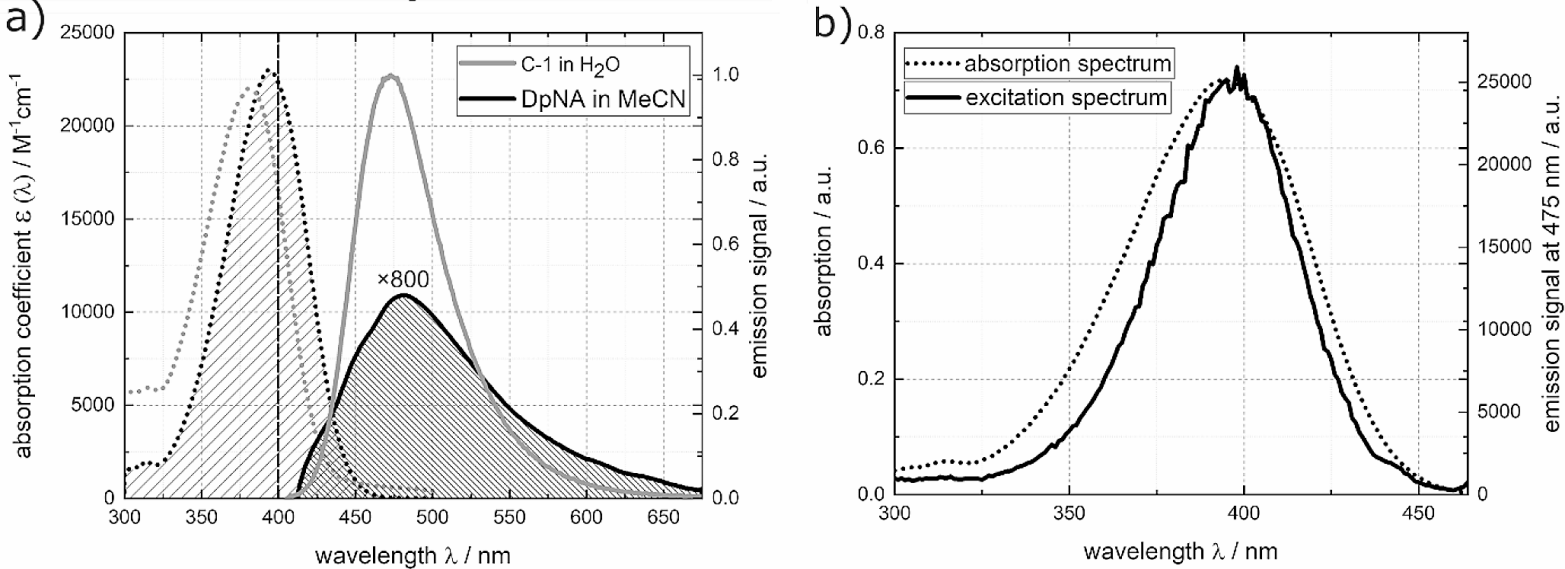
(**a**) Absorption (coefficient, black dotted line) and fluorescence (smoothed black solid line) spectra of DpNA in acetonitrile. Absorption (coefficient, gray dotted line scaled according to ref. [65]) and fluorescence (gray solid line) spectra of the reference dye C-1 in water are included. The excitation wavelength at 400 nm is marked in the absorption spectra. The emission spectra were recorded with constant wavelength bandpass (5 nm). The fluorescence spectra are scaled such that their integrals are proportional to their respective fluorescence quantum yields. For the sake of comparison, the fluorescence spectrum of DpNA was multiplied by a factor of 800. The relevant ranges used for the Strickler-Berg analysis are highlighted in the absorption and emission spectra. (**b**) Fluorescence excitation spectrum of DpNA in comparison with its absorption spectrum. For the excitation spectrum the signal was probed at 475 nm

DpNA and its derivative 4-nitroaniline were shown to undergo ultrafast IC with sub-picosecond time constants [60, 61]. Thus, a fluorescence quantum yield *Ф*_fl._ of the desired magnitude is to be expected. DpNA in acetonitrile exhibits a structureless absorption band lowest in transition energy peaking around 394 nm (see Fig. 2). The fluorescence spectrum peaks at 480 nm. The spectra converted into the transition dipole representation [41, 62] reveal that the mirror-image relationship holds approximately (see Fig. S1 in the Online Resource). The fluorescence excitation spectrum slightly deviates from the properly scaled absorption spectrum shown in Fig. 2b for DpNA in acetonitrile. Its absorption coefficient ɛ_max_ was determined to be (2.30 ± 0.08)× 10^4^ M^− 1^cm^− 1^, which is in line with the previously reported value of 2.42× 10^4^ M^−1^cm^−1^ [63]. For the relative determination of the fluorescence quantum yield, DpNA dissolved in acetonitrile was excited close to the maximum at 400 nm (see Fig. 2). The resulting fluorescence signal was compared to the one of C-1 in water. The fluorescence quantum yield based on the relative approach ${\varPhi }_{fl}^{rel}$ was computed using Eq. (4) [8]

4 $${\varPhi }_{fl}^{rel}={\varPhi }_{fl}^{r}\frac{\int {S}_{fl}^{s}\left(\lambda \right)d\lambda }{\int {S}_{fl}^{r}\left(\lambda \right)d\lambda }\frac{{A}^{r}}{{A}^{s}}{\left(\frac{{n}^{s}}{{n}^{r}}\right)}^{2}.$$

Here, ${\varPhi }_{fl}^{r}$ is the fluorescence quantum yield of the reference, $\int {S}_{fl}^{s,r}\left(\lambda \right)d\lambda$ are the spectral integrals of the fluorescence for sample and reference. ${A}^{s,r}<0.05$ are the absorptions of sample and reference at the excitation wavelength, and ${n}^{s,r}$ denotes the refractive index of the solvent of the sample or the reference. Values complied in ref. [52, 64] were inserted. With these inputs, a yield ${\varPhi }_{fl}^{rel}$ of (5.12±0.06)× 10^−5^ results for DpNA in acetonitrile.

Using the spectra depicted in Fig. 2, a Strickler-Berg analysis was conducted. In this analysis, the radiative rate constant ${k}_{rad}$ is obtained from spectral integrals (covering a part) of the absorption spectrum and the fluorescence spectrum (Eqs. (5, 6)) [40, 41],

5 $${k_{rad}} = \frac{{8\pi {\rm{ln}}\left( {10} \right){c_0}{n^2}}}{{{N_A}}}{\left\langle {{{\tilde v}^{ - 3}}} \right\rangle ^{ - 1}}\int {\frac{{\varepsilon \left( {\tilde v} \right)d\tilde v}}{{\tilde v}}},$$

6 $${\left\langle {{{\tilde v}^{ - 3}}} \right\rangle ^{ - 1}} = \frac{{\int {{S_{fl}}} \left( {\tilde v} \right)d\tilde v}}{{\int {{{\tilde v}^{ - 3}}} {S_{fl}}\left( {\tilde v} \right)d\tilde v}}.$$

Here, ${c}_{0}$ is the speed of light, $n$ the refractive index of the solvent, and ${N}_{A}$ Avogadro´s number. The factor ${\left\langle {{{\tilde v}^{ - 3}}} \right\rangle ^{ - 1}}$ accounts for the cubic dependence of the spontaneous emission on the wavenumber $\stackrel{\sim}{\nu }$. Its evaluation involves integrals covering the fluorescence spectrum ${S}_{fl}\left(\stackrel{\sim}{\nu }\right)$ as a function of the wavenumber $\stackrel{\sim}{\nu }.$ The fluorescence spectra ${S}_{fl}^{\lambda }\left(\lambda \right)$were recorded as a function of the wavelength $\lambda$ and with a constant wavelength bandpass (5 nm). For the conversion to wavenumber axis, the spectrum ${S}_{fl}^{\lambda }\left(\lambda \right)$ was multiplied by the wavelength $\lambda$ squared, ${S}_{fl}\left(\stackrel{\sim}{\nu }\right)\sim{S}_{fl}^{\lambda }\left(\lambda \right){\lambda }^{2}$ [8]. The molar decadic absorption $\varepsilon \left( {\tilde v} \right)$ as a function of the wavenumber $\stackrel{\sim}{\nu }$ enters the integral $\int {\frac{{\varepsilon \left( {\tilde v} \right)d\tilde v}}{{\tilde v}}}$. It is crucial that this integral only covers the part of the spectrum $\varepsilon \left( {\tilde v} \right)$ associated with the transition to the lowest excited singlet state. The respective range is marked in Fig. 2. The respective evaluation affords a radiative rate constant ${k}_{rad}$ of (1.72 ± 0.08)× 10^8^ s^−1^.

**Fig. 3:**
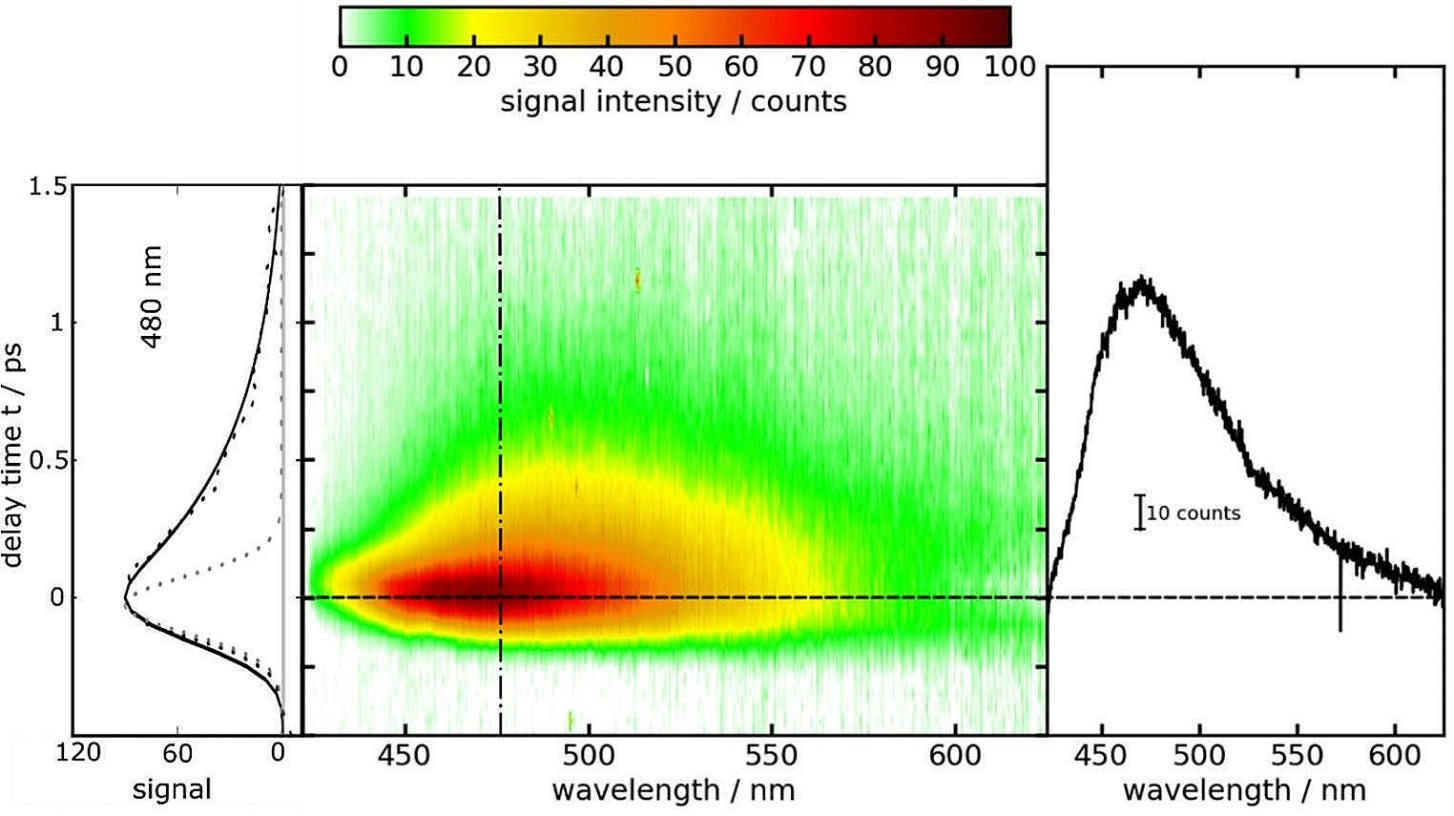
Femtosecond transient fluorescence on DpNA in acetonitrile (∼ 0.5 mM) as a function of detection wavelength *λ* and delay time *t*. The solution was excited at 400 nm. In the central contour representation, reddish hue represents large fluorescence signals. One representative time trace (480 nm) as well as a fit are shown on the left. The dotted gray line represents the IRF

In the fluorescence Kerr gating experiment, a solution of DpNA dissolved in acetonitrile was excited with femtosecond pulses centered at 400 nm (Fig. 3). Time resolved spectra closely match the shape of the steady state one, with a peak around 480 nm. Within one picosecond, almost all the emission signal has vanished (Fig. 3). The experimental results were subject to a global analysis using a single-exponential convoluted with the IRF as a trial function (see Experimental section). The procedure affords a fluorescence lifetime ${\tau }_{fl.}$ of 590 ± 190 fs. In a previous study a time constant of 630 fs was reported [61]. Multiplying this lifetime with the radiative rate constant determined above results in a fluorescence quantum yield ${\varPhi }_{fl}^{SB}$of (1.01 ± 0.3)× 10^− 4^ (see Eq. (2)). This value is approximately twice the yield ${\varPhi }_{fl}^{rel}$ determined by the relative approach. Due to this discrepancy, DpNA was discarded as a reference.

Semi-empirical quantum chemical computations [66] and transient absorption experiments [61] performed by Ernsting et al. can rationalize this discrepancy. For the closely related molecule 4-nitroaniline, these computations predict an ultrafast (< 100 fs) decrease of the oscillator strength *f* and thereby the radiative rate constant *k*_*rad*_ after photo-excitation. In transient absorption experiments with a time resolution of ∼ 50 fs, which compares to ∼ 250 fs in the fluorescence experiments reported here, such an ultrafast decrease was observed for DpNA in acetonitrile [61]. Notably, a decrease by a factor of ∼ 0.5 is observed. Such a non-Condon effect is not incorporated into the (standard) Strickler-Berg approach. If the reduction of the radiative rate constant *k*_*rad*_ by a factor of 0.5 is taken into account, the yields ${\varPhi }_{fl}^{rel}$ and ${\varPhi }_{fl}^{SB}$ match. Despite this, DpNA was discarded since for the other chromophores described below, matching values were obtained without such complications.

### Blue Region of the UV/Vis Spectrum – dT in Water

**Fig. 4:**
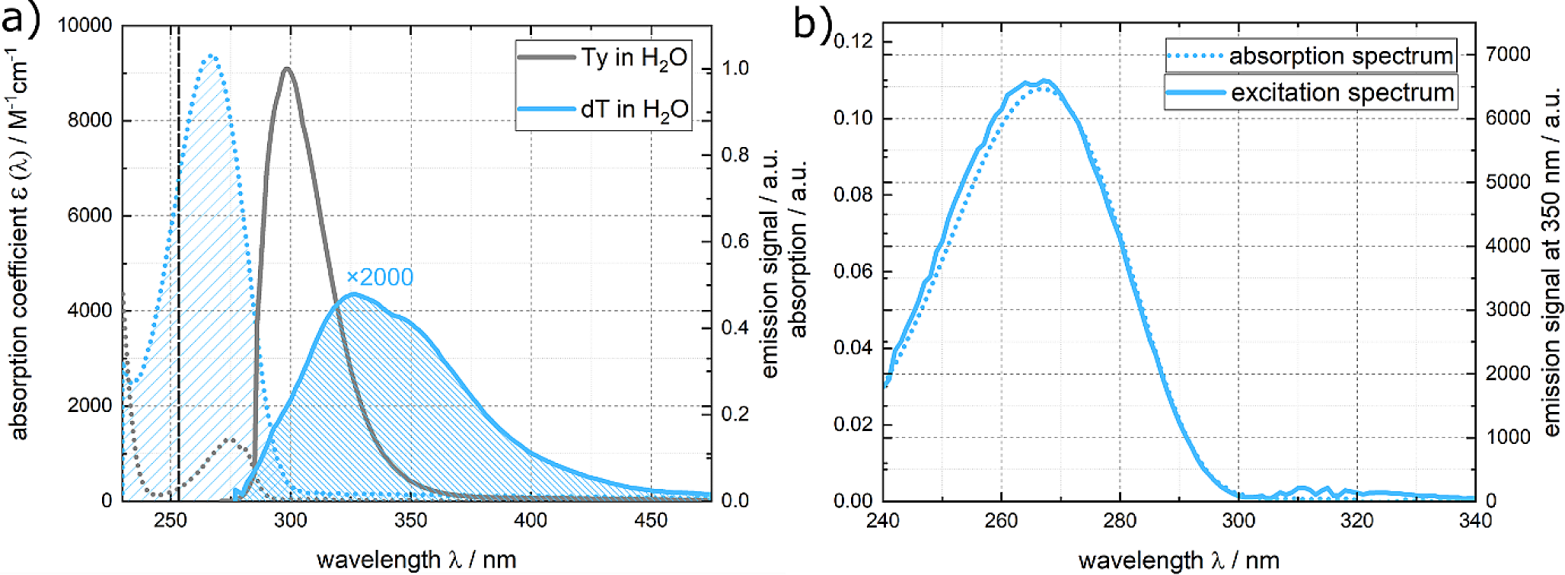
(**a**) Absorption (coefficient, blue dotted line) and fluorescence (smoothed blue solid line) spectra of dT in water. Absorption (coefficient, gray dotted line scaled according to ref. [71]) and fluorescence (gray solid line) spectra of the reference dye Ty in water are included. The excitation wavelength at 255 nm is marked in the absorption spectra. The emission spectra were recorded with constant wavelength bandpass (5 nm). The fluorescence spectra are scaled such that their integrals are proportional to their respective fluorescence quantum yields. For the sake of comparison, the fluorescence spectrum of dT was multiplied by a factor of 2000. The relevant ranges used for the Strickler-Berg analysis are highlighted in the absorption and emission spectra. (**b**) Fluorescence excitation spectrum of dT in comparison with its absorption spectrum. For the excitation spectrum the signal was probed at 350 nm

The photophysics of thymidine has been extensively studied due to its fundamental role as a DNA building block [46, 67, 68]. These studies have shown that dT undergoes ultrafast internal conversion in a couple of 100 fs [69, 70]. Previous studies provide values for the fluorescence quantum yield of dT in the order of 10^− 4^ [45, 46]. In this study, we aimed to reproduce these reported values. dT in water exhibits a structureless absorption band lowest in transition energy peaking around 267 nm (see Fig. 4). The fluorescence spectrum peaks at 330 nm. The spectra converted into the transition dipole representation reveal an approximate mirror-image relationship (see Fig. S2 in the Online Resource). The fluorescence excitation spectrum overlays favorably with the properly scaled absorption spectrum (Fig. 4b). Its absorption coefficient ɛ_max_ was determined to be (9.4 ± 0.1)× 10^3^ M^− 1^cm^− 1^, which is somewhat smaller than the previously reported value of 9.7× 10^3^ M^− 1^cm^− 1^ [46]. For a relative determination of the fluorescence quantum yield, dT dissolved in water was excited close to the maximum at 255 nm (see Fig. 4). The resulting fluorescence signal was compared to that of Ty in water. Using Eq. (4), the relative fluorescence quantum yield ${\varPhi }_{fl}^{rel}$ was calculated, relying on values compiled in ref. [54, 64]. From these inputs, a yield ${\varPhi }_{fl}^{rel}$ of (1.3 ± 0.09)× 10^− 4^ for dT in water was obtained.

Using the spectra shown in Fig. 4, a Strickler-Berg analysis was performed. The relevant ranges for this analysis are highlighted in Fig. 4. This evaluation yielded a radiative rate constant *k*_*rad*_ of (2.30 ± 0.03)× 10^8^ s^− 1^ (see Eq. (5)).

**Fig. 5:**
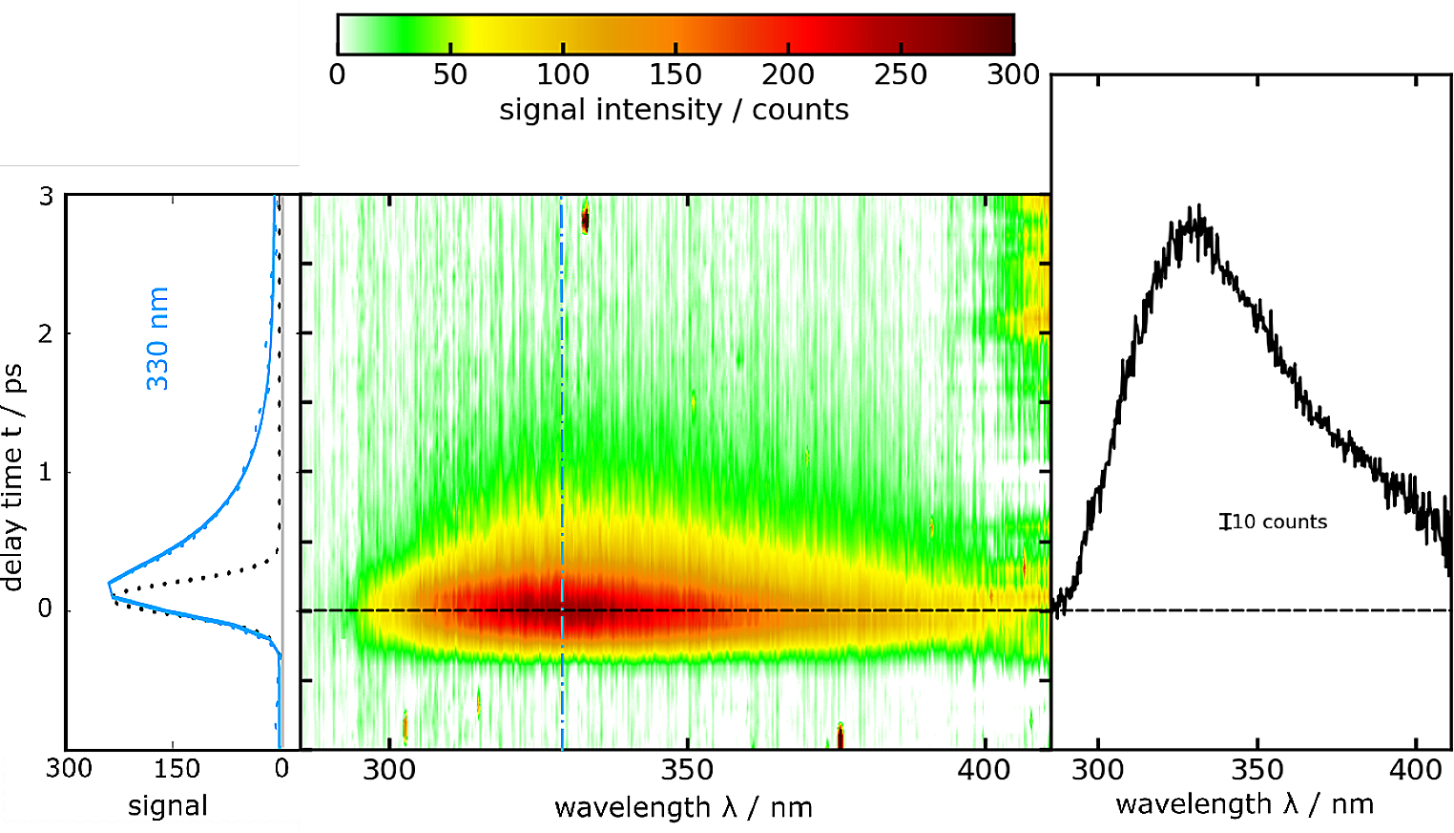
Femtosecond transient fluorescence on dT in water (∼ 1 mM) as a function of detection wavelength *λ* and delay time *t*. The solution was excited at 266 nm. In the central contour representation, reddish hue represents large fluorescence signals. One representative time trace (330 nm) as well as a single-exponential fit are shown on the left. The dotted black line represents the IRF

**Fig. 6:**
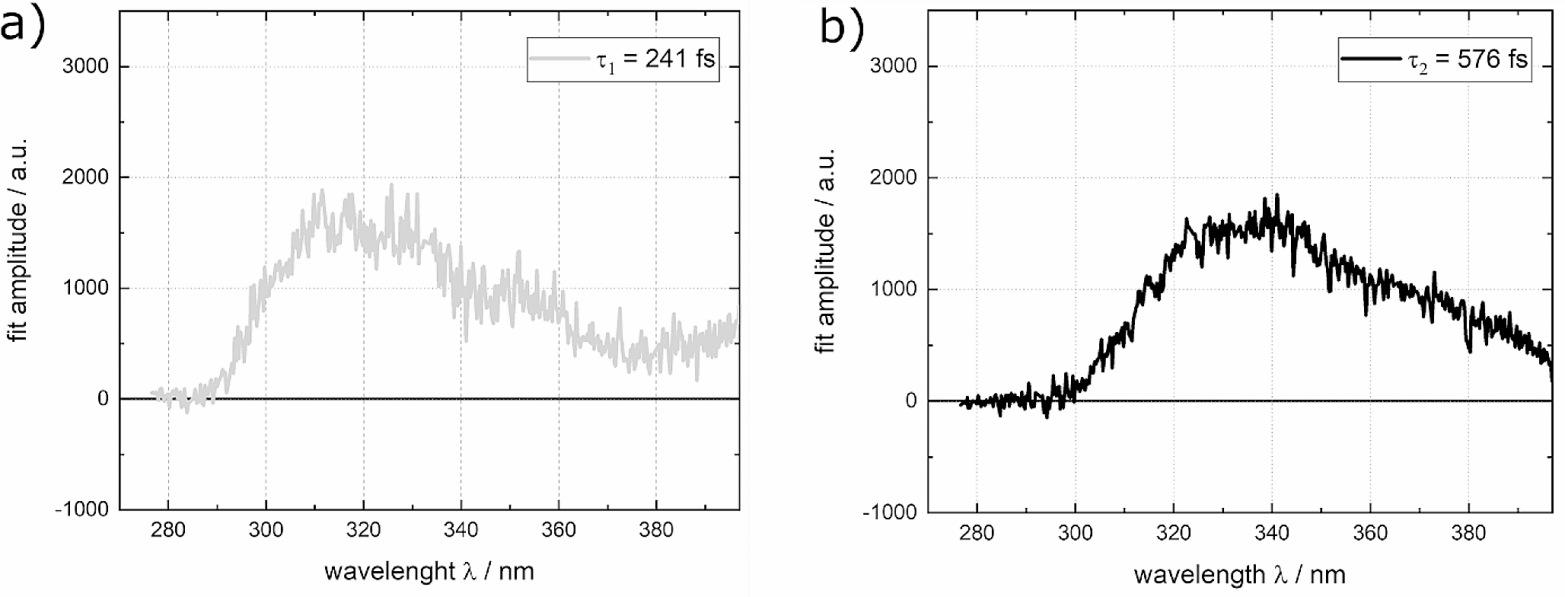
Decay associated spectra (DAS) retrieved from the measurement on dT in water depicted in Fig. 5 using a bi-exponential trial function

A solution of dT in water was excited using femtosecond pulses centered at 266 nm and the resulting emission signal was probed using fluorescence Kerr gating (Fig. 5). The time resolved spectra closely resemble the shape of the steady state spectrum, exhibiting a peak around 330 nm. Within one picosecond, almost all the emission signal has vanished (Fig. 5). To determine the fluorescence lifetime ${\tau }_{fl.}$ of dT, a global fit of the data was performed. This analysis employed both single- and bi-exponential trial functions, convoluted with the IRF (see Experimental section). The single-exponential fit for dT in water resulted in a fluorescence lifetime ${\tau }_{fl.}$ of 480 ± 140 fs. The bi-exponential fit afforded lifetimes of ${\tau }_{1}\approx$240 fs and ${\tau }_{2}\approx$ 580 fs (Fig. 6). An average fluorescence lifetime $\langle {\tau }_{fl.}\rangle$ was derived using the Eq. (7),

7 $$\left\langle {{\tau _{fl}}} \right\rangle = \frac{{\int {DA{S_1}} \cdot {\tau _1} + \int {DA{S_2}} \cdot {\tau _2}}}{{\int {DA{S_1}} + \int {DA{S_2}} }}.$$

Here, $\int {DAS}_{1}$ and $\int {DAS}_{2}$ represent the spectral integrals of both decay associated spectra which are depicted in Fig. 6. This equation (Eq. 7) yields an average fluorescence lifetime of $\langle {\tau }_{fl.}\rangle =$ 408 ± 190 fs, which is somewhat smaller than the time constant obtained from the single-exponential fit. Time constants in a similar (470–700 fs) range have been reported in prior studies [46, 69, 72]. Multiplying the lifetime obtained from the single-exponential fit with the above radiative rate constant results in a fluorescence quantum yield ${\varPhi }_{fl}^{SB}$of (1.11 ± 0.3)× 10^− 4^ (see Eq. (2)). Inserting the average fluorescence lifetime into the same equation (Eq. 2), results in a marginally lower fluorescence quantum yield ${\varPhi }_{fl}^{SB}$ of (0.938 ± 0.4)× 10^− 4^. However, both ${\varPhi }_{fl}^{SB}$ values obtained here closely align with the yield ${\varPhi }_{fl}^{rel}$ determined by the relative approach.

### Green Region of the UV/Vis Spectrum – DBM in Ethanol

**Fig. 7:**
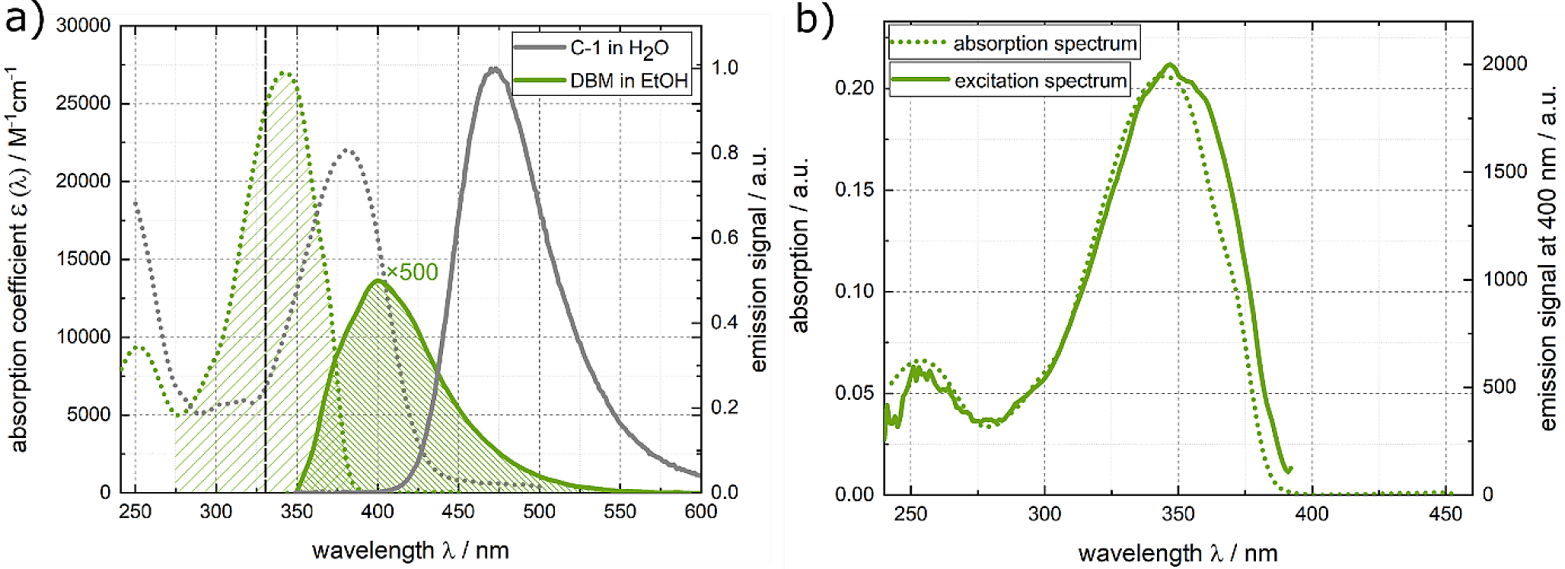
(**a**) Absorption (coefficient, green dotted line) and fluorescence (smoothed green solid line) spectra of DBM in ethanol. Absorption (coefficient, gray dotted line scaled according to ref. [65]) and fluorescence (gray solid line) spectra of the reference dye C-1 in water are included. The excitation wavelength at 330 nm is marked in the absorption spectra. The emission spectra were recorded with constant wavelength bandpass (5 nm). The fluorescence spectra are scaled such that their integrals are proportional to their respective fluorescence quantum yields. For the sake of comparison, the fluorescence spectrum of DBM was multiplied by a factor of 500. The relevant ranges used for the Strickler-Berg analysis are highlighted in the absorption and emission spectra. (**b**) Fluorescence excitation spectrum of DBM in comparison with its absorption spectrum. For the excitation spectrum the signal was probed at 400 nm

DBM undergoes ultrafast intramolecular proton transfer [73] upon photo-excitation. While the fluorescence quantum yield for DBM has not been quantified before, its fluorescence lifetime was found to be in the sub-picosecond range [47]. DBM in ethanol displays a structureless absorption band lowest in transition energy peaking around 340 nm (see Fig. 7). The fluorescence spectrum peaks at 400 nm. The spectra converted into the transition dipole representation reveal an approximate mirror-image relationship (see Fig. S3 in the Online Resource). The fluorescence excitation spectrum closely aligns with the properly scaled absorption spectrum for DBM in ethanol, as shown in Fig. 7b. The peak absorption coefficient ɛ_max_ of (2.71 ± 0.04)× 10^4^ M^− 1^cm^− 1^ at 340 nm determined here is close to a value of 2.5× 10^4^ M^− 1^cm^− 1^ reported earlier [74]. To determine the fluorescence quantum yield of DBM relatively, a solution of DBM in ethanol was excited close to the maximum at 330 nm (see Fig. 7). The observed fluorescence signal was compared to the fluorescence of C-1 in water. Employing Eq. (4) and incorporating reference values [52, 64], the relative fluorescence quantum yield ${\varPhi }_{fl}^{rel}$ was determined to be (6.88 ± 0.05)× 10^− 5^ for DBM in ethanol.

Utilizing the spectra presented in Fig. 7, a Strickler-Berg analysis was conducted. The relevant ranges for this analysis are highlighted in Fig. 7. From this analysis, a radiative rate constant *k*_*rad*_ of (2.37 ± 0.04)× 10^8^ s^− 1^ was derived (see Eq. (5)).

**Fig. 8:**
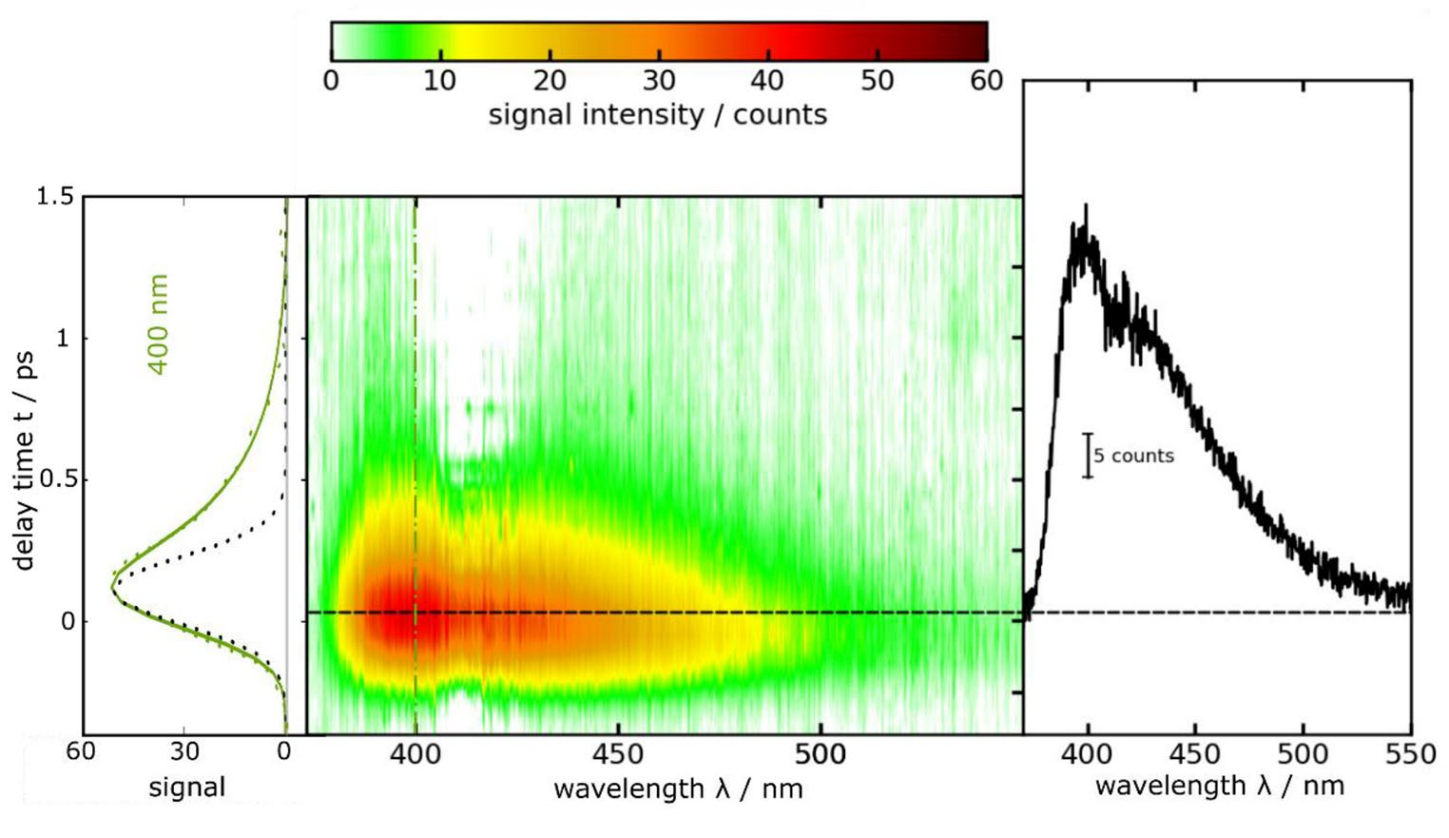
Femtosecond transient fluorescence on DBM in ethanol (∼ 1.3 mM) as a function of detection wavelength *λ* and delay time *t*. The solution was excited at 266 nm. In the central contour representation, reddish hue represents large fluorescence signals. One representative time trace (400 nm) as well as a fit are shown on the left. The dotted black line represents the IRF

In the fluorescence Kerr gating experiment, femtosecond pulses centered at 266 nm were used to excite a solution of DBM dissolved in ethanol (Fig. 8). The time resolved spectra were found to closely resemble the shape of the steady state spectrum, with a peak observed around 400 nm. Within half a picosecond, nearly all the emission signal has vanished (Fig. 8). The results of the experiment were subject to a global analysis, where a single-exponential convoluted with the IRF was employed as a trial function (see Experimental Section). This analysis afforded a fluorescence lifetime ${\tau }_{fl.}$ of 290 ± 80 fs. A previous study reported a time constant of 240 fs [47]. By multiplying this lifetime by the radiative rate constant *k*_*rad*_, a fluorescence quantum yield ${\varPhi }_{fl}^{SB}$of (6.92 ± 1.9)× 10^− 5^ was calculated (see Eq. (2)).

### Red Region of the UV/Vis Spectrum – MG in Water

**Fig. 9:**
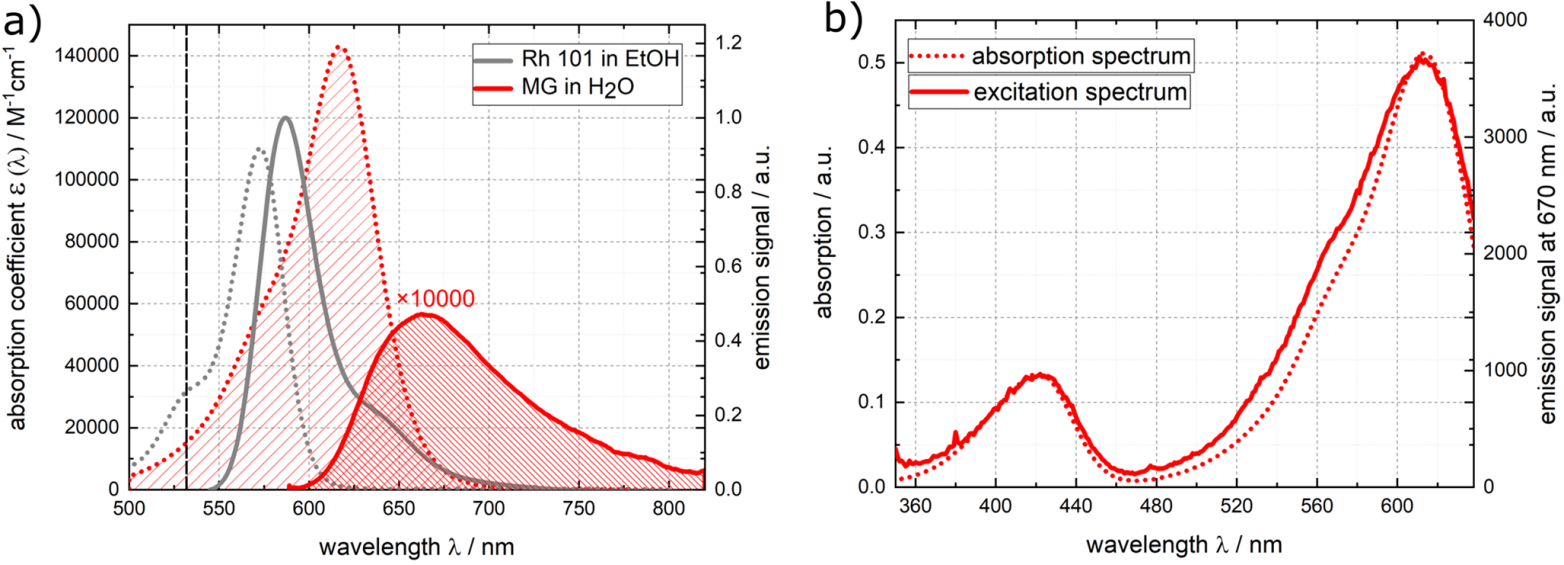
(**a**) Absorption (coefficient, red dotted line) and fluorescence (smoothed red solid line) spectra of MG in water. Absorption (coefficient, gray dotted line scaled according to ref. [82]) and fluorescence (gray solid line) spectra of the reference dye Rh 101 in ethanol are included. The excitation wavelength at 535 nm is marked in the absorption spectra. The emission spectra were recorded with constant wavelength bandpass (5 nm). The fluorescence spectra are scaled such that their integrals are proportional to their respective fluorescence quantum yields. For the sake of comparison, the fluorescence spectrum of MG was multiplied by a factor of 10,000. The relevant ranges used for the Strickler-Berg analysis are highlighted in the absorption and emission spectra. (**b**) Fluorescence excitation spectrum of MG in comparison with its absorption spectrum. For the excitation spectrum the signal was probed at 670 nm

MG, a triphenylmethane dye, exhibits pronounced visible absorption bands and has a very low fluorescence quantum yield ($\le$10^−4^) in low-viscosity liquid solutions [75, 76]. Prior studies have shown that MG undergoes ultrafast internal conversion [49, 76–79]. MG in water exhibits an band lowest in transition energy peaking around 618 nm (see Fig. 9). The fluorescence spectrum peaks at 670 nm. An approximate mirror-image relationship is disclosed upon converting the spectra into the transition dipole representation (see Fig. S4 in the Online Resource). The fluorescence excitation spectrum for MG in water closely matches the properly rescaled absorption spectrum (see Fig. 9b). The peak absorption coefficient ɛ_max_ of (1.43 ± 0.01)× 10^5^ M^−1^cm^−1^ at 618 nm determined here is in line with the value of 1.40× 10^5^ M^−1^ cm^−1^ reported earlier [80, 81]. For a relative determination of its fluorescence quantum yield, MG dissolved in water was excited at 535 nm (see Fig. 9). The resulting fluorescence signal was compared to the one of Rh 101 in ethanol. The fluorescence quantum yield based on the relative approach ${{\Phi }}_{fl}^{rel}$ was computed using Eq. (4). Values compiled in ref. [55, 64] were inserted. With these inputs, a yield ${\varPhi }_{fl}^{rel}$ of (9.67 ± 0.5)× 10^−5^ results for MG in water.

Using the spectra depicted in Fig. 9, a Strickler-Berg analysis was conducted. The ranges are marked in Fig. 9. The evaluation affords a radiative rate constant *k*_*rad*_ of (2.18 ± 0.05)× 10^8^‌ s^− 1^ (see Eq. (5)).

**Fig. 10:**
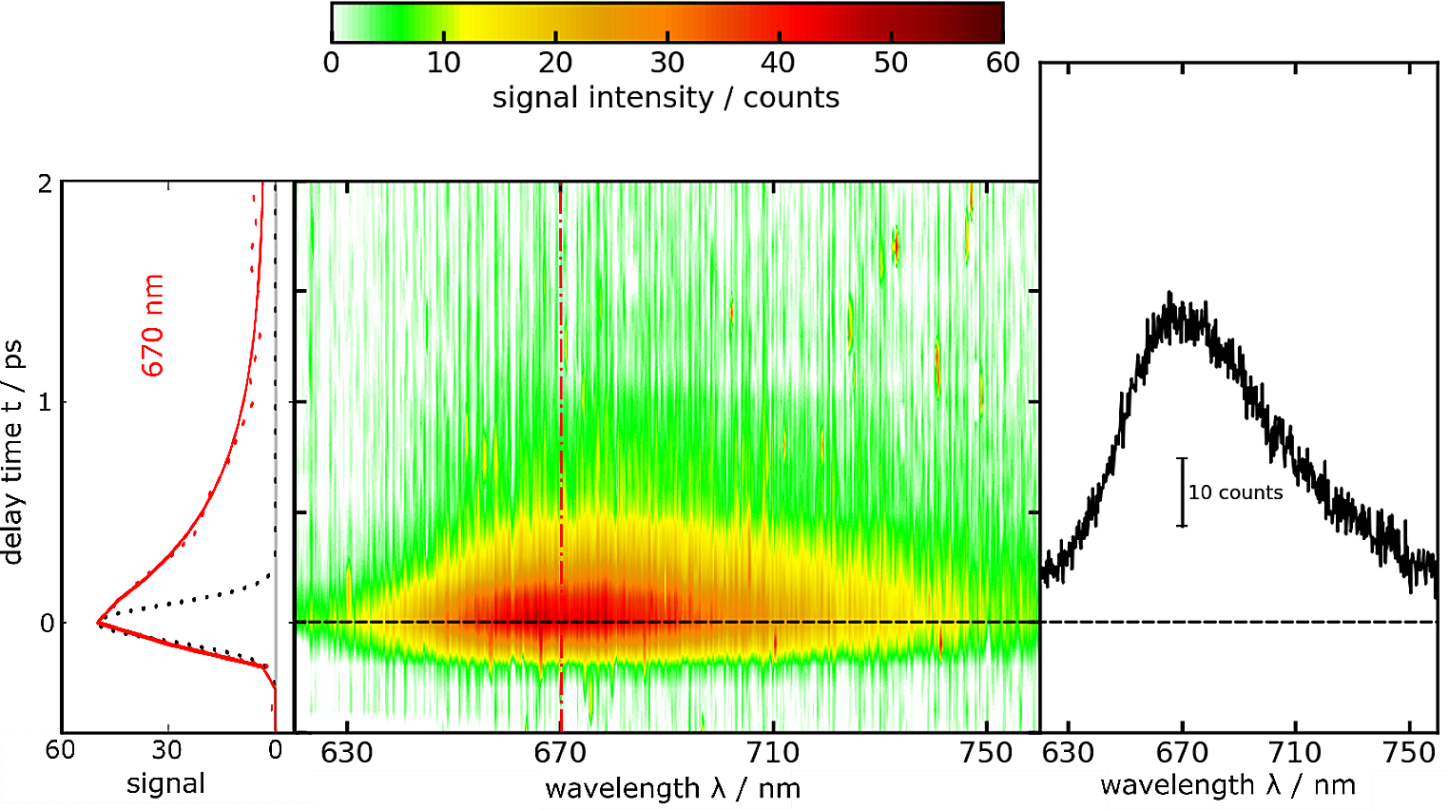
Femtosecond transient fluorescence on MG in water (∼ 70 μM) as a function of detection wavelength *λ* and delay time *t*. The solution was excited at 580 nm. In the central contour representation, reddish hue represents large fluorescence signals. One representative time trace (670 nm) as well as a fit are shown on the left. The dotted black line represents the IRF

In the fluorescence Kerr gating experiment, femtosecond pulses with a center wavelength of 580 nm were utilized to excite a solution of MG in water (Fig. 10). The resulting time resolved spectra closely matched the steady state spectrum in shape, peaking around 670 nm. Almost the entire emission signal disappeared within one picosecond (Fig. 10). A global analysis was conducted utilizing a single-exponential function convoluted with the IRF as the trial function. This procedure yielded a fluorescence lifetime ${\tau }_{fl.}$ of 450 ± 160 fs. Prior studies reported time constants in a similar (520–660 fs) range [48–51]. By multiplying this lifetime by the radiative rate constant *k*_*rad*_, a fluorescence quantum yield ${{\Phi }}_{fl}^{SB}$ of (9.86 ± 3.5)× 10^− 5^ was calculated (see Eq. (2)).

## Conclusion

In this study, we propose three compounds as new references for determining small fluorescence quantum yields in the UV/Vis spectral range, with yields ranging from 10^− 5^ to 10^− 4^. These compounds are thymidine in water for the blue region, dibenzoylmethane in ethanol for the green region, and malachite green chloride in water for the red region of the spectrum. Each of these compounds is easily handled, photostable, commercially available, and demonstrates a mirror-image symmetry between its absorption and fluorescence spectra. This symmetry indicates the involvement of the same electronic states in absorption and emission processes, thereby supporting the application of the Strickler-Berg relation. Furthermore, the fluorescence excitation spectra of all compounds closely align with their respective absorption spectra, confirming that the observed emissions originate from the compounds under study. The fluorescence quantum yields determined using both the relative and time resolved techniques, exhibit satisfactory agreement within their respective error margins (see Table 1). However, the error margins for the fluorescence quantum yields obtained via the time resolved technique are slightly larger, mainly due to the error in the fluorescence lifetimes (Table 1). Our findings for the fluorescence quantum yield of dT, obtained through both methods, align well with previous studies, suggesting the reliability of our results for the other compounds. It is worth noting that no value for DBM and only an upper boundary for the yield *Ф*_fl._ of MG were reported previously. Here, the fluorescence quantum yields for both compounds were precisely determined.

**Table 1:** Fluorescence quantum yields determined in this work in comparison with the earlier reports

	${\varvec{\varPhi }}_{\varvec{f}\varvec{l}}^{\varvec{r}\varvec{e}\varvec{l}}$ from relative method	${\varvec{\varPhi }}_{\varvec{f}\varvec{l}}^{\varvec{S}\varvec{B}}$ from k_rad_ × ${\tau }_{fl.}$	${\varvec{\varPhi }}_{\varvec{f}\varvec{l}}$ from prior studies
dT in water	(1.3 ± 0.09)× 10^− 4^	(1.11 ± 0.3)× 10^− 4 a^ (0.938 ± 0.4)× 10^− 4 b^	1.32× 10^-4^ [46], 1× 10^-4^ [45]
DBM in ethanol	(6.88 ± 0.05)× 10^− 5 c^	(6.92 ± 1.9)× 10^− 5^	Almost non-fluorescent in solution [73, 83, 84]
MG in water	(9.67 ± 0.5)× 10^− 5^	(9.86 ± 3.5)× 10^− 5^	$<$10^− 4^ [75], $\sim$10^− 4^ [76]

a Based on a single-exponential fit

b Based on a bi-exponential fit

c No error margins for the yield ${\varPhi }_{fl}^{r}$ were available

## Electronic Supplementary Material

Below is the link to the electronic supplementary material.

Supplementary Material 1

## Data Availability

The datasets generated during and/or analysed during the current study are available from the corresponding author on reasonable request.
