# Supplementary material for: References for Small Fluorescence Quantum Yields
Source: J Fluoresc. 2024 May 15;35(5):3253–66. doi: 10.1007/s10895-024-03729-2 (PMC12095370; doi:10.1007/s10895-024-03729-2)
Supplement: Supplementary file 1 — Supplementary Material 1 [file 10895_2024_3729_MOESM1_ESM.pdf]

Online Resource:

References for Small Fluorescence Quantum Yields

*Mahbobeh Morshedi[1], Simon L. Zimmermann[1], David Klaverkamp[1], and Peter Gilch[*1]*

[1]Institut für Physikalische Chemie, Heinrich-Heine-Universität Düsseldorf, Universitätsstr. 1, 40225 Düsseldorf, Germany

Corresponding Author
*E-mail: gilch@hhu.de

Transition dipole representation

Mirror-image relationships between absorption and fluorescence spectra are best investigated relying on the transition dipole representation [1, 2]. This representation corrects absorption and fluorescence signals for trivial frequency dependencies. To arrive at this representation, absorption and fluorescence signals recorded as a function of the wavelength λ were plotted as a function of the wavenumber $\tilde{\nu}$. This conversion of the fluorescence spectrum entailed a λ^2 factor (see main text). In the transition dipole representation, the absorption signals are plotted as $\varepsilon(\tilde{\nu})/\tilde{\nu}$ and the fluorescence ones as $S_{fl}(\tilde{\nu})/\,\tilde{\nu}^3$ (see Fig. S1, S2, S3, S4).

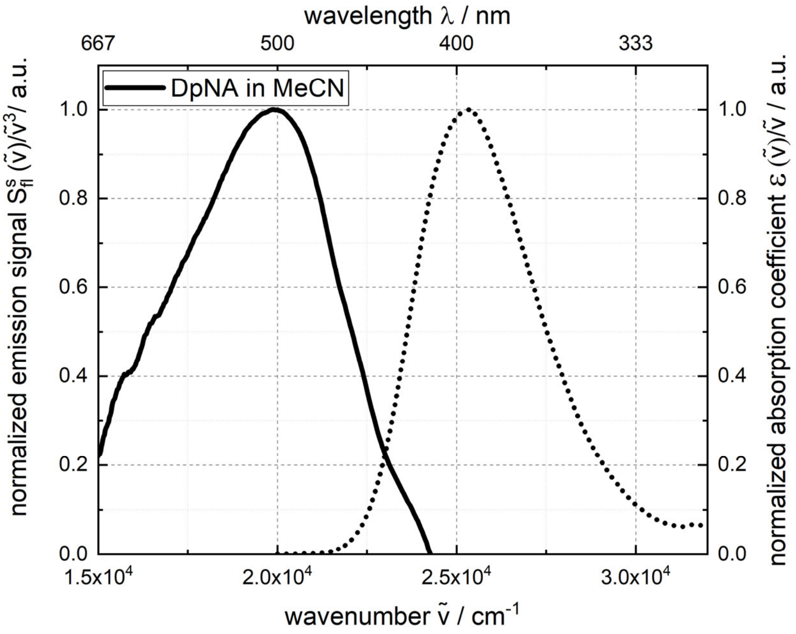

Fig. S1 Absorption (coefficient, black dotted line) and fluorescence (smoothed black solid line) spectra of DpNA in acetonitrile in transition dipole representation. The spectra were converted into the transition dipole representation and then normalized. To record the fluorescence spectrum, the excitation wavelength was set to 400 nm

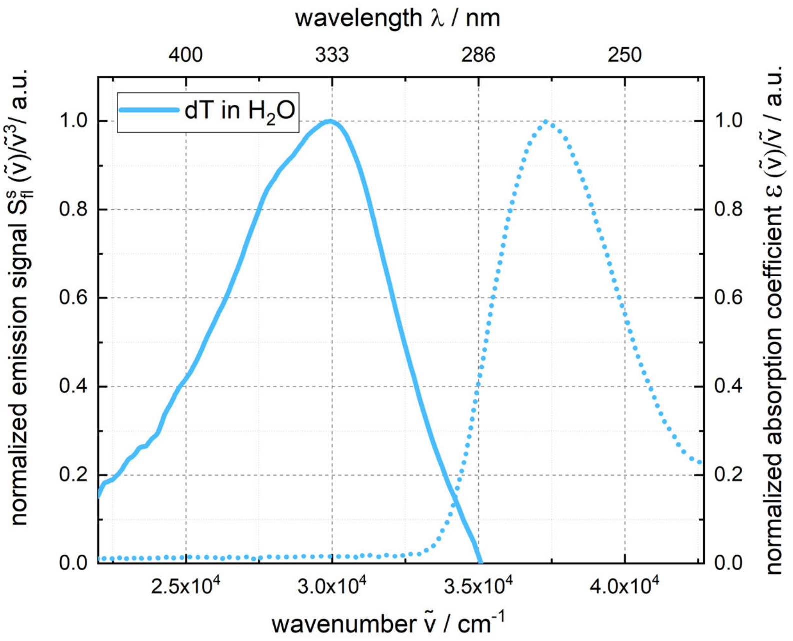

Fig. S2 Absorption (coefficient, blue dotted line) and fluorescence (smoothed blue solid line) spectra of dT in water in transition dipole representation. The spectra were converted into the transition dipole representation and then normalized. To record the fluorescence spectrum, the excitation wavelength was set to 255 nm

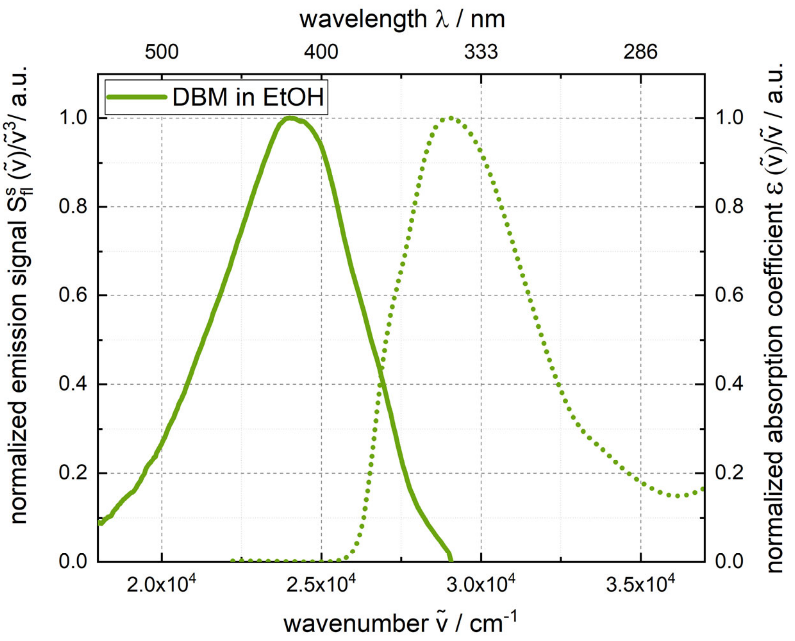

Fig. S3 Absorption (coefficient, green dotted line) and fluorescence (smoothed green solid line) spectra of DBM in ethanol in transition dipole representation. The spectra were converted into the transition dipole representation and then normalized. To record the fluorescence spectrum, the excitation wavelength was set to 330 nm

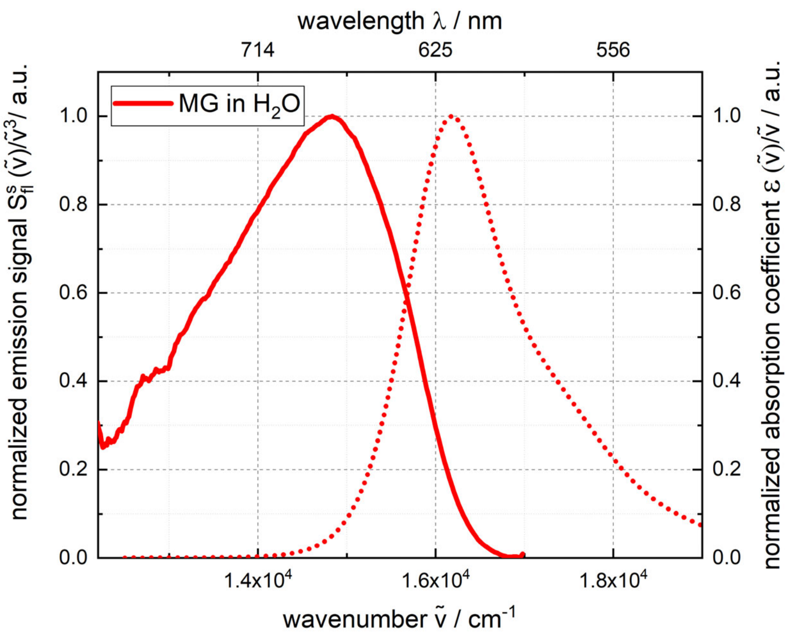

Fig. S4 Absorption (coefficient, red dotted line) and fluorescence (smoothed red solid line) spectra of MG in water in transition dipole representation. The spectra were converted into the transition dipole representation and then normalized. To record the fluorescence spectrum, the excitation wavelength was set to 535 nm

References

1. Angulo G, Grampp G, Rosspeintner A (2006) Recalling the appropriate representation of electronic spectra. Spectrochimica Acta Part A: Molecular Biomolecular Spectroscopy 65:727-731. https://doi.org/10.1016/j.saa.2006.01.007

2. Parson W W (2007) Modern optical spectroscopy. Springer, Berlin.
